# Effect of Oral Choline Alfoscerate on Patients with Keratoconjunctivitis Sicca

**DOI:** 10.3390/nu12051526

**Published:** 2020-05-23

**Authors:** Jin Ju Choi, Jin Sun Hwang, Young Joo Shin

**Affiliations:** Department of Ophthalmology, Hallym University Medical Center, Hallym University College of Medicine, Seoul 150-719, Korea; 920128@hallym.or.kr (J.J.C.); hotsayme@naver.com (J.J.H.)

**Keywords:** choline alfoscerate, keratoconjunctivitis sicca, tear break-up time, dry eye symptoms

## Abstract

Keratoconjunctivitis sicca (KCS) or dry eye is a disease characterized by ocular surface symptoms. This study aimed to investigate the effectiveness of oral choline alfoscerate (CA) administration as a treatment for KCS. The medical records of dry eye patients who were refractory to topical eyedrops and then took oral CA were reviewed. Results of tear break-up time (TBUT), fluorescein ocular surface staining score (FSS), and tear secretion by the Schirmer test (STT) were analyzed. The results of the ocular surface disease index (OSDI), visual analog pain score (VAS), reporting of the severity and frequency of symptoms, and the modified Standardized Patient Evaluation of Eye Dryness (SPEED) questionnaire were also analyzed. The records of 47 patients were analyzed for this study. The mean age was 62.8 ± 9.3 years, and the patients included 9 males and 38 females. TBUT, OSDI, and VAS significantly improved after CA administration compared to before (*p* < 0.05, paired *t*-test). After CA administration, symptom frequency and impact on life improved (*p* < 0.05, paired *t*-test). No significant change in photophobia or FSS was identified. In conclusion, oral CA administration was effective in improving tear stability and alleviating symptoms of KCS.

## 1. Introduction

Keratoconjunctivitis sicca (KCS) or dry eye is a disease characterized by ocular surface symptoms, including grittiness, pain, burning, stinging, foreign body sensation (FBS), and photophobia. However, dry eye is a disease in which the amount of tears present on the ocular surface is insufficient because of excessive evaporation or low tear secretion [1]. KCS symptoms result from a reduction in both the quantity and quality of tear production. Therapy for KCS includes artificial tear application and topical administration of the bioactive drugs diquafosol and cyclosporine A (CsA) [2]. Punctal plugs can be used to increase tear retention [2]. Despite improvement of the tear film and ocular surface characteristics, KCS symptoms often do not respond to treatment [3]. Recently, a neuropathic mechanism has been implicated as one cause for this [3].

Aging is an identified risk factor for KCS [4] as is cholinergic innervation loss [5]. However, significant cholinergic cell loss during aging as a cause of KCS has been challenged [6]. Acetylcholine is the primary parasympathetic neurotransmitter [7]. The parasympathetic innervation of the lacrimal apparatus is supplied by the petrosal nerve [8]. The secretion of proteins, electrolytes, and water by the lacrimal gland depends on neurotransmitters (acetylcholine and norepinephrine) released by the activation of either the parasympathetic or sympathetic nerves [8]. The meibomian glands and conjunctival goblet cells have muscarinic receptors [9,10].

Choline is an essential nutrient, and it can be acquired from the diet [11]. Choline alfoscerate (CA), also called alpha glycerylphosphorylcholine, is a natural choline compound and a parasympathomimetic acetylcholine precursor [12]. Phosphatidylcholine has been proposed to potentiate the synthesis of acetylcholine [12]. Phosphatidylcholine is thought to have therapeutic potential in patients with peripheral neuropathy and brain injury because of its antioxidant properties and ability to modulate microglial activity [13]. The analgesic effect of phosphatidylcholine is induced by stimulation of muscarinic receptors, and its anti-neuropathic effect is mediated via stimulation of nicotinic receptors [14]. Choline supplementation could improve the symptoms of KCS; however, this has not been previously reported. In this study, we investigated the effect of oral choline alfoscerate administration on patients with KCS.

## 2. Methods

This retrospective cohort study was approved by the Hallym University Medical Center Institutional Review Board and was carried out following the principles of the Declaration of Helsinki. Between January 2015 and May 2019, patients with KCS symptoms refractory to topical treatment were prescribed oral administration of CA. Records of these patients were reviewed; patients were included in the study if they met the following inclusion criteria: Older than 45 years of age; presence of clinical signs and symptoms of KCS; presence of symptoms refractory to treatment with artificial tears, bioactive drugs, and punctal plug; and clinical examination variable assessments and survey completions prior to two weeks and/or six weeks after commencing oral CA. Topical treatments were prescribed continuously. 

Clinical endpoints included tear break-up time (TBUT), lid hyperemia, ocular surface disease index (OSDI), visual analog pain score (VAS), symptom scores, fluorescein ocular surface staining score (FSS), and Schirmer tear test (STT). Patients were asked to provide a VAS and complete a modified Standardized Patient Evaluation of Eye Dryness (SPEED) questionnaire. TBUT was measured using fluorescein staining strips. FSS was graded using the Oxford grading scale [15]. OSDI was calculated after patient OSDI questionnaire completion [16]. VAS was assessed using a segmental numerical technique [17]. 

A modified SPEED was used to assess the frequency and severity of each symptom, including dryness, foreign body sensation, coldness, eye fatigue, pain, and photophobia [18]. The SPEED questionnaire was scored for both frequency of symptoms (0 = no symptoms, 1 = sometimes, 2 = often, and 3 = constant) and severity of symptom/impact on daily life (0 = no symptoms, 1 = tolerable but not uncomfortable, 2 = uncomfortable but does not interfere with my day, 3 = bothersome and interferes with my day, and 4 = intolerable and unable to perform my daily tasks).

### Statistical Analysis

Only one eye in each patient was evaluated. A mixed-effects model analysis was performed using Graphpad Prism 8.2 (GraphPad Software, San Diego, CA) to examine time-related changes of parameters. Paired *t*-tests were used to examine differences in the parameters between each time point using Statistical Package for Social Sciences (SPSS) software (version 24, PSS, Inc, Chicago, IL, USA). Statistical significance was defined as *p* < 0.05. A Wilcoxon signed-rank test instead of the paired *t*-test was used for the nonparametric statistical analysis.

## 3. Results

In total, 47 eyes of 47 patients were included. Nine males and 38 females with a mean age of 62.8 ± 9.3 years were enrolled. Forty-four patients returned two weeks after commencing oral CA, and 32 patients returned six weeks after commencing oral CA. Before commencing oral CA, mean TBUT was 5.9 ± 2.7 s, FSS was 0.27 ± 0.61, lid hyperemia was 1.5 ± 0.9, tear secretion was 8.8 ± 7.0 mm, OSDI was 1.1 ± 24.3, VAS was 3.2 ± 2.5, impact of dry eye symptoms on daily life was 2.8 ± 0.9, and daily frequency was 2.7 ± 1.0 (Table 1). The severity and frequency of each symptom before taking oral CA are described in Table 2 and Table 3.

**Figure 1:**
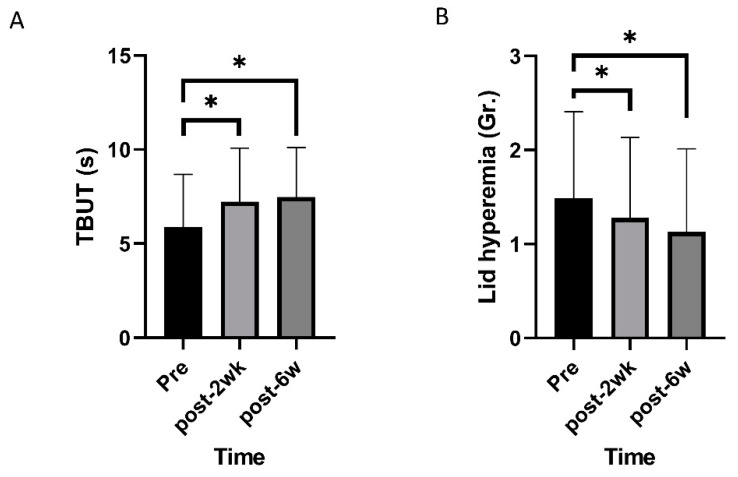
Changes of objective signs of dry eye disease over time. (**A**) Tear break-up time (TBUT) increased over time. (**B**) Lid hyperemia grade decreased over time. * statistically significant.

**Figure 2:**
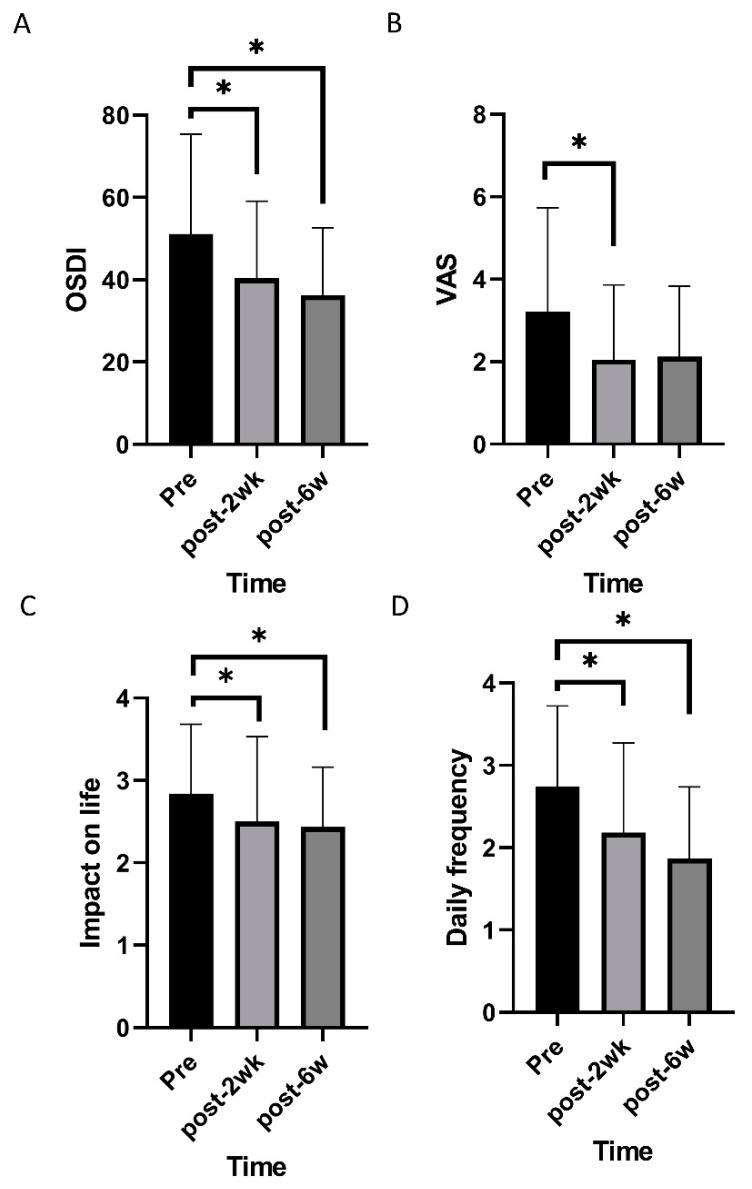
Changes of subjective symptoms of dry eye disease over time. (**A**,**B**) Ocular surface disease index (OSDI) and visual analogue pain score (VAS) decreased over time. (**C**,**D**) Impact of dry eye symptoms on life and daily frequency of dry eye symptoms decreased over time. * statistically significant.

**Figure 3:**
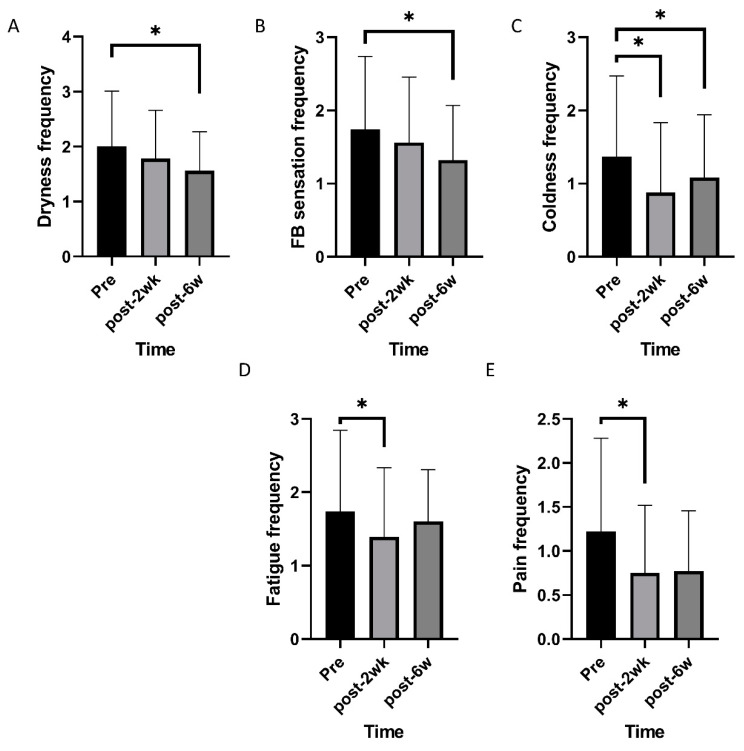
Changes of frequency of individual symptoms over time. (**A**,**B**) Frequencies of dryness and foreign body sensation decreased over time. (**C**–**E**) Frequencies of coldness, eye fatigue, and pain changed over time. * statistically significant.

**Figure 4:**
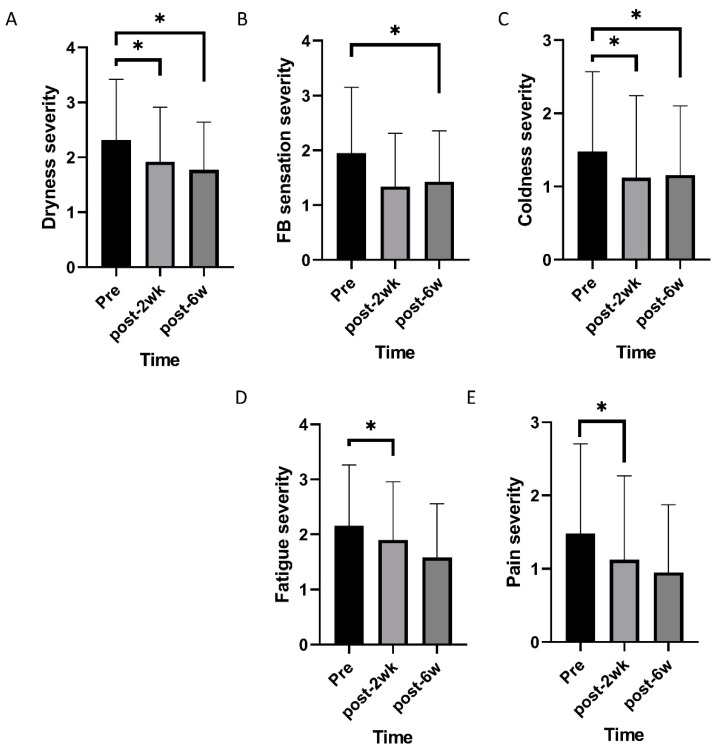
Changes of severity of individual symptoms over time. (**A**–**C**) Severity of dryness, foreign body sensation, and coldness decreased over time. (**D**) Severity of eye fatigue and pain changed over time. * statistically significant.

Mixed effects model analysis identified an improvement in TBUT and lid hyperemia after commencing oral CA (*p* < 0.001 and 0.005; Figure 1). OSDI, VAS, impact of dry eye symptoms, and daily frequency of dry eye symptoms decreased overtime (*p* < 0.001, 0.019, 0.022, and <0.001; Figure 2). Daily frequency of dryness, coldness, and pain decreased overtime (*p* = 0.049, 0.004, and 0.010; Figure 3). Severity of dryness, FBS, coldness, and fatigue decreased over time (*p* = 0.027, 0.013, 0.016, and 0.026; Figure 4).

Two weeks after commencing oral CA, TBUT, OSDI, and VAS were improved compared to the baseline (*p* = 0.001, 0.003, and 0.004, paired *t*-test; Table 1), and lid hyperemia, impact of dry eye symptoms on life, and daily frequency was reduced compared to the baseline (*p* = 0.014, 0.034, and 0.009, Wilcoxon signed-rank test; Table 1). There were no significant changes in FSS and STT. After commencing CA, scores for daily frequency of coldness, fatigue, and pain were reduced (*p* = 0.003, 0.010, and 0.003, Wilcoxon signed-rank test; Table 2), and scores for the severity of dryness, coldness, fatigue, and pain were improved (*p* = 0.008, 0.019, 0.004, and 0.044, Wilcoxon signed-rank test; Table 3).

Six weeks after commencing oral CA, TBUT and OSDI had improved compared to the baseline (*p* = 0.001, and 0.004, paired *t*-test; Table 1), and lid hyperemia, impact of dry eye symptoms on life, and daily frequency were reduced compared to the baseline (*p* = 0.016, 0.011, and 0.002, Wilcoxon signed-rank test; Table 1). FSS and STT were not statistically different from the pretreatment values. Each symptom was also evaluated. Daily frequency of dryness, FB sensation, and coldness were reduced compared to pretreatment values (*p* = 0.001, 0.026, and 0.036, Wilcoxon signed-rank test; Table 2). Severity of dryness, FB sensation, and coldness were improved compared to pretreatment values (*p* = 0.007, 0.006, and 0.021, Wilcoxon signed-rank test; Table 3). 

## 4. Discussion

Choline has been used to produce acetylcholine, a neurotransmitter in the parasympathetic nervous system [19]. In this retrospective study, we investigated whether oral CA administration improved the symptoms of KCS. Improvement in TBUT, lid hyperemia, symptoms associated with pain, dryness, sensation, and fatigue were identified. Three potential mechanisms may explain the apparent effect of CA administration. First, CA is a surfactant, which may act as an emulsifier, stabilizing the water and lipid layers of the tear film. Next, choline supplementation has been reported to attenuate immune inflammation and suppress oxidative stress [19], which contribute to the pathophysiology of KCS. Cholinergic modulation of the immune response presents a new approach to treat inflammation [20]. Acetylcholine-stimulated nicotinic receptors on macrophages cause a concentration-dependent inhibition of proinflammatory cytokine release [21]. Furthermore, choline is a precursor of the parasympathetic neurotransmitter acetylcholine. Muscarinic receptors are present in the cornea conjunctiva [10] and meibomian glands and play a role in wound healing and secretion [9]. Cholinergic transmission profoundly modifies the perception of pain [22]. Cholinergic inhibition of spinal nociceptive transmission [23] leads to the analgesic effects of exogenously administrated cholinergic agonists in neuropathic pain [24]. Antinociceptive effects of cholinergic agonists are associated with gamma-aminobutyric acid-ergic (GABAergic) signaling and nicotinic and muscarinic modulation of nociceptive transmission. Notably, we did not identify any reduction in photophobia in patients enrolled in this study. Another approach may be necessary to reduce the photophobic symptoms of KCS. FSS was not changed because FSS was too low at the baseline. Up to 83.0% of patients showed no FSS before CA treatment. STT was not changed, although CA is a parasympathomimetic drug. CA may have an effect on KCS mainly through an anti-neuropathic effect in the central nervous system and an enhancement of tear stability as a surfactant in tears. CA may need to be combined with a cholinesterase inhibitor to have a sufficient effect on tear secretion [25].

Polyunsaturated fatty acids supplementation, including omega-3 and omega-6 fatty acids, is well known as a nutritional therapy for KCS [26,27,28,29,30], although the main treatment of KCS is topical treatments [31]. Dietary omega-3 and omega-6 fatty acids supplementation improves the symptoms and inflammation of KCS [32,33,34,35,36]. Omega-6 and omega-3 fatty acids are essential polyunsaturated fatty acids that cannot be synthesized in the body and must be obtained from the diet [32]. Although a higher dietary intake of omega-3 fatty acids is associated with a lower prevalence of dry eye in females [28], a high ratio of omega-6/omega-3 fatty acids (>15:1) was associated with a higher than two-fold prevalence of dry eye [28]. The ideal balance of omega-3/omega-6 fatty acids has been suggested to be 1:2.3 [27]. Omega-3 fatty acids include alpha-linolenic acid (ALA), eicosapentaenoic acid (EPA), and docosahexaenoic acid (DHA) [27]. Oral supplementation of omega-3 fatty acids showed a decrease in the tear evaporation rate, an improvement in dry eye symptoms, and an increase in tear secretion [32,34]. CA might help stabilize the tear film in addition to omega-3 supplementation.

Limitations to this study include its retrospective nature, which can lead to several biases and may not identify some risks or contributing factors, and its small sample size. A sufficiently powered future prospective study could contribute further support for the administration of oral choline for the treatment of KCS. In conclusion, this study identified that oral CA administration is effective in securing tear stability and alleviating the symptoms of dry eye.

## Figures and Tables

**Table 1:** Signs and symptoms change before and after taking oral choline alfoscerate.

	Before Taking Oral Choline Alfoscerate	Two Weeks after Taking Oral Choline Alfoscerate	Six Weeks after Taking Oral Choline Alfoscerate
N	47	44	32
Age (y)	62.8 ± 9.3	62.9 ± 9.1	61.9 ± 8.9
Male:female	9:38	8:36	5:27
TBUT (s)	5.9 ± 2.7	7.2 ± 2.9 *	7.5 ± 2.6 *
FSS	0.27 ± 0.61	0.26 ± 0.53	0.27 ± 0.57
Lid hyperemia	1.5 ± 0.9	1.3 ± 0.9 †	1.1 ± 0.9 †
OSDI	51.1 ± 24.3	40.4 ± 18.7 *	36.2 ± 16.4 *
VAS	3.2 ± 2.5	2.1 ± 1.8 *	2.1 ± 1.7
Impact on the daily life	2.8 ± 0.9	2.5 ± 1.0 †	2.4 ± 0.8 †
Daily frequency	2.7 ± 1.0	2.2 ± 1.1 †	1.9 ± 0.9 †

TBUT = tear break-up time; FSS = fluorescein staining score; OSDI = ocular surface disease index; VAS = visual analogue scale. * < 0.05 by paired *t*-test.; † < 0.05 by Wilcoxon signed-rank-test.

**Table 2:** Changes of each symptom frequency before and after taking oral choline alfoscerate.

	Before Taking Oral Choline Alfoscerate	Two Weeks after Taking Oral Choline Alfoscerate	Six Weeks after Taking Oral Choline Alfoscerate
Dryness	2.0 ± 1.0	1.8 ± 0.9	1.6 ± 0.7 †
FB sensation	1.8 ± 1.0	1.6 ± 0.9	1.3 ± 0.7 †
Coldness	1.4 ± 1.1	0.9 ± 1.0 †	1.1 ± 0.9 †
Fatigue	1.7 ± 1.1	1.4 ± 0.9 †	1.6 ± 0.7
Pain	1.2 ± 1.1	0.7 ± 0.7 †	0.8 ± 0.7
Photophobia	1.2 ± 1.2	1.2 ± 1.1	1.1 ± 1.0

† < 0.05 by Wilcoxon signed-rank-test.

**Table 3:** Changes of each symptom severity before and after taking oral choline alfoscerate.

	Before Taking Oral Choline Alfoscerate	Two Weeks after Taking Oral Choline Alfoscerate	Six Weeks After Taking Oral Choline Alfoscerate
Dryness	2.3 ± 1.1	1.9 ± 1.0 †	1.8 ± 0.9 †
FB sensation	2.2 ± 1.1	1.9 ± 1.1	1.5 ± 1.0 †
Coldness	1.5 ± 1.1	1.1 ± 1.1 †	1.2 ± 0.9 †
Fatigue	1.9 ± 1.2	1.4 ± 1.0 †	1.5 ± 1.0
Pain	1.5 ± 1.2	1.1 ± 1.1 †	1.0 ± 0.9
Photophobia	1.3 ± 1.2	1.2 ± 1.1	1.1 ± 1.0

† < 0.05 by Wilcoxon signed-rank-test.
